# hiPSC-Based Model of Prenatal Exposure to Cannabinoids: Effect on Neuronal Differentiation

**DOI:** 10.3389/fnmol.2020.00119

**Published:** 2020-07-06

**Authors:** Cláudia C. Miranda, Tiago Barata, Sandra H. Vaz, Carla Ferreira, Alexandre Quintas, Evguenia P. Bekman

**Affiliations:** ^1^Department of Bioengineering and iBB—Institute for Bioengineering and Biosciences, Instituto Superior Técnico, Universidade de Lisbon, Lisbona, Portugal; ^2^Instituto de Medicina Molecular, João Lobo Antunes, Faculdade de Medicina da Universidade de Lisboa, Lisbon, Portugal; ^3^Molecular Pathology and Forensic Biochemistry Laboratory, CiiEM, Campus Universitário Quinta da Granja, Monte da Caparica, Caparica, Portugal; ^4^Forensic and Psychological Sciences Laboratory Egas Moniz, Campus Universitário Quinta da Granja, Monte da Caparica, Caparica, Portugal; ^5^The Discoveries Centre for Regenerative and Precision Medicine, Lisbon, Portugal

**Keywords:** phytocannabinoids, synthetic cannabinoids, hiPSC, neuronal differentiation, Δ^9^-THC, CBD, EG-018, THJ-018

## Abstract

Phytocannabinoids are psychotropic substances ofcannabis with the ability to bind endocannabinoid (eCB) receptors that regulate synaptic activity in the central nervous system (CNS). Synthetic cannabinoids (SCs) are synthetic analogs of Δ^9^-tetrahydrocannabinol (Δ^9^-THC), the psychotropic compound of cannabis, acting as agonists of eCB receptor CB_1_. SC is an easily available and popular alternative to cannabis, and their molecular structure is always changing, increasing the hazard for the general population. The popularity of cannabis and its derivatives may lead, and often does, to a child’s exposure to cannabis both *in utero* and through breastfeeding by a drug-consuming mother. Prenatal exposure to cannabis has been associated with an altered rate of mental development and significant changes in nervous system functioning. However, the understanding of mechanisms of its action on developing the human CNS is still lacking. We investigated the effect of continuous exposure to cannabinoids on developing human neurons, mimicking the prenatal exposure by drug-consuming mother. Two human induced pluripotent stem cells (hiPSC) lines were induced to differentiate into neuronal cells and exposed for 37 days to cannabidiol (CBD), Δ^9^-THC, and two SCs, THJ-018 and EG-018. Both Δ^9^-THC and SC, at 10 μM, promote precocious neuronal and glial differentiation, while CBD at the same concentration is neurotoxic. Neurons exposed to Δ^9^-THC and SC show abnormal functioning of voltage-gated calcium channels when stimulated by extracellular potassium. In sum, all studied substances have a profound impact on the developing neurons, highlighting the importance of thorough research on the impact of prenatal exposure to natural and SC.

## Introduction

Phytocannabinoids, such as Δ^9^-THC, are substances found in cannabis that can bind to the endocannabinoid (eCB) system receptors, which regulate a variety of physiological processes in the human body, such as synaptic activity in the central nervous system (CNS), and analgesic and metabolic effects in the peripheric nervous system, PNS (Pertwee, 2008; Wu et al., 2011; Metz and Stickrath, 2015). Cannabinoid receptors 1 (CB_1_) and 2 (CB_2_), are expressed in the developing brain and there is growing evidence supporting the role of these receptors in neural progenitor proliferation and modulation of neuronal maturation and specification (Galve-Roperh et al., 2013). The expression of these receptors in the fetal and adult human brain was also reported (reviewed in Galve-Roperh et al., 2009). Prenatal exposure to cannabinoids acting as agonists of CB_1_ and CB_2_ receptors can produce long-lasting effects on eCB signaling affecting motor activity, verbal development, nociception, drug-seeking behavior and other processes (reviewed in Broyd et al., 2016; Richardson et al., 2016; Grant et al., 2018). Due to their lipophilic nature, phytocannabinoids can easily permeate cellular membranes passing from drug-consuming mothers’ bloodstream into foetal tissues (Grotenhermen, 2003). Prenatal exposure to cannabis has been associated with lower weight at birth and a higher risk of newborn morbidity (Hurd et al., 2005; Metz and Stickrath, 2015). In two extended cohort studies, altered rate of mental development and significant changes in nervous system functioning were consistently found (Fried, 2002; Smith et al., 2004; Gray et al., 2005). However, the cellular mechanisms underlying cannabinoid effects on human neural development are still poorly known. Thus, there is an urgent need for a better understanding of the impact of these substances on human brain development, especially due to the contemporary trend of increasing cannabis use.

Besides this trend, a new problem has emerged in the form of synthetic analogs of Δ^9^-tetrahydrocannabinol (Δ^9^-THC). Synthetic cannabinoids (SCs) are a group of Novel Psychoactive Substances with similar properties to Δ^9^-THC that appeared on the drug market usually sold as herbal blends (United Nations Office on Drugs and Crime, The Challenge of New Psychoactive Substances; 2013). The SCs usually show a higher affinity to the CB_1_ receptor and elicit a stronger and long-lasting effect on brain cells when compared to Δ^9^-THC. Also, many of these substances are easily available on the internet and thus escape the control of authorities. SCs use is associated with more severe side effects and intoxications, with both neurologic symptoms and acute organ toxicity observed (Schoeder et al., 2018). Two SCs used in this study are derivatives of JWH-018, the first indole-based potent CB_1,_ and CB_2_ receptor agonist (Atwood et al., 2010) with a toxicity profile diverging from that of Phyto-CBs (Grant et al., 2018). THJ-018 is a 2nd generation SC, with CB_1_ binding affinity of 5.84 nM and a CB_2_ binding affinity of 4.57 nM (Hess et al., 2016). EG-018 is a 3rd generation SC, which replaced THJ-018, with an affinity to CB_1_ of 7.17 nM and CB_2_ of 2.22 nM (Schoeder et al., 2018). This study aims to address the impact of SCs with higher affinity than Δ^9^-THC for CB_1_ and/or CB_2_ on developing human brain cells using neural differentiation of human-induced pluripotent stem cells (hiPSCs). The effect of the non-psychotropic component of cannabis, CBD, that binds the CB_2_ receptor and was shown to act as a negative allosteric modulator of CB_1_ (Laprairie et al., 2015) was also evaluated. Controlled aggregation of hiPSCs in neural-inducing medium allows recapitulating the initial steps of the self-organizing neural tube and subsequent progenitor proliferation and production of neurons of forebrain identity (Shi et al., 2012; Miranda et al., 2015). This system represents a simple and reproducible *in vitro* model that allows assessing the effect of continuous exposure to cannabinoid on the development of human brain cells at molecular, cellular, and functional levels. Two hiPSC lines were induced into neural differentiation and treated with CBD, Δ^9^-THC and two synthetic Δ^9^-THC analogues, THJ-018 and EG-18. Our results indicate that all four substances have profound impact on the differentiation, maturation and functioning of developing CNS neurons, providing a new evidence for the importance of thorough research of the impact of prenatal exposure to cannabis and its synthetic analogues.

## Materials and Methods

### Maintenance of Human iPSCs

Human-induced pluripotent stem cells (hiPSCs), Gibco^®^ Human Episomal iPSC line derived from CD34^+^ cord blood (iPSC6.2, Burridge et al., 2011) and F002.1A.13 (TCLab—Tecnologias Celulares para Aplicação Médica, Unipessoal, Lda.) were routinely cultured on Matrigel^TM^ (1:100, Corning)-coated plates using mTeSR^TM^1 medium (StemCell Technologies). Cells were passaged 1:5 using EDTA every 5 days (Beers et al., 2012).

### Neural Commitment and Differentiation

hiPSCs were induced towards neural commitment as 3D aggregates using a modified dual SMAD inhibition protocol (Miranda et al., 2015) and allowed to achieve functional differentiation using the recently described BrainPhys medium (Bardy et al., 2015). Briefly, cells were incubated with 10 μM ROCK inhibitor (ROCKi, Y-27632, StemGent) for 1 h at 37°C and then treated with accutase for 5 min at 37°C. Cells were seeded in microwell plates (AggreWell^TM^, StemCell Technologies) at a density of 1.0 × 10^6^ cells/ml to generate aggregates averaging a diameter of 150 μm using mTeSR^TM^1 supplemented with 10 μM ROCKi for 24 h. After 24 h of culture inside microwells, mTeSR1 medium was replaced by 1:1 N2 and B27 medium, as previously described (Shi et al., 2012). The medium was replaced daily and supplemented with 10 μM SB431542 (SB, Sigma) and 100 nM LDN193189 (LDN, StemGent) for 9 days, followed by a 3-day period without SB431542 and LDN19318.

On day 12 aggregates were recovered from the microwells, gently dissociated with EDTA, and plated onto poly-L-ornithine (15 μg/ml, Sigma) and Laminin (20 μg/ml, Sigma)-coated plates at a density of 200,000 cells/cm^2^ in N2B27 medium. Twenty-four hours after replating, the medium was replaced by N2B27 supplemented with 20 ng/ml bFGF from day 13 to day 15 of differentiation. From day 15 onwards, the medium was changed every other day without bFGF supplementation.

### Exposure to Cannabinoids and Neuronal Maturation

On day 19 of the differentiation protocol, cells were gently detached from the plates and re-plated with 1:3 splitting in the same conditions, for neuronal differentiation and drug treatment. From day 19 to day 30 of differentiation CBD, Δ^9^-THC and two different SCs, EG-018 and THJ-018 were added to the medium at every medium change at a concentration of 10 μM in ethanol, except CBD, which was added at a 1–10 μM concentration. Untreated and vehicle (0.01% ethanol)-treated cultures were used as a control. At day 30 gentle replating was performed once again and the cultures were allowed to mature in complete BrainPhys^TM^ Neuronal Medium (StemCell Technologies)—supplemented with NeuroCult^TM^ SM1 Neuronal Supplement (StemCell Technologies), N2 Supplement-A (StemCell Technologies), Recombinant Human Brain-Derived Neurotrophic Factor (BDNF, PeproTech, 20 ng/ml), Recombinant Human Glial-Derived Neurotrophic Factor (GDNF, PeproTech, 20 ng/ml), dibutyryl cAMP (1 mM, Sigma), and ascorbic acid (200 nM, Sigma). One-third of the medium volume was changed every 3 days.

### Immunostaining

Cells were fixed with 4% (v/v) paraformaldehyde (PFA; Sigma) and stained according to a previously described protocol (Miranda et al., 2015). MAP2 (Sigma, 1:500), glial fibrillary acidic protein (GFAP, Abcam, 1:200), Synaptophysin (SYN; Abcam, 1:200), ZO-1 (Novex, 1:100), SOX2 (R&D, 1:200), PAX6 (Covance, 1:400), NESTIN (R&D, 1:400), Ki-67 (Abcam, 1:100), HuC/D (Thermo Fischer Scientific, 1:100), activated CASPASE3 (pCASP3, Cell Signaling, 1:400), were used as primary antibodies whereas goat anti-mouse IgG Alexa Fluor–488 or 546 (1:500, Invitrogen), goat anti-rabbit IgG Alexa Fluor–488 or 546 (1:500, Invitrogen) were used as secondary antibodies. Fluorescence images were acquired with Zeiss LSM 710 Confocal Laser Point-Scanning Microscope using 20× and 63× objectives and integrated density were calculated for each channel using ImageJ software. The ratio between integrated density for the marker of interest and nuclear counterstaining with DAPI was calculated for each image. For each staining, the same acquisition settings were applied for all images.

### Real-Time (RT)-PCR

For quantitative analysis, total RNA was extracted at different time-points of differentiation and treatments using the High Pure RNA Isolation Kit (Roche), according to the manufacturer’s instructions. Total RNA was converted into complementary cDNA with Transcriptor High Fidelity cDNA Synthesis Kit (Roche) using 500 ng of RNA. Relative gene expression was evaluated using 10 ng of cDNA and 250 μM of each primer.

Expression levels were analyzed using SYBR^®^ green chemistry, with primers for *GAPDH, PAX6, MAP2, NESTIN, GFAP, GAD67*, and *VGLUT1* from Silva et al. (2020). Primers for *CNR1* and *CNR2* were from Stanslowsky et al. (2017).

All PCR reactions were done in triplicate, using the ViiA^TM^ 7 RT-PCR Systems (Applied BioSystems). Fold change was calculated using the 2^−ΔCt^ method, using GAPDH as the reference gene. Log2 normalized expression values of the average fold-change were used for ClustVis analysis of pluripotency and neural genes (Metsalu and Vilo, 2015).

### Single-Cell Calcium Imaging

To analyze the intracellular variations of Ca^2+^ by single-cell calcium imaging (SCCI), cells were re-plated on Glass Bottom Cell Culture Dish (Nest) previously coated with poly-L-ornithine (15 μg/ml, Sigma) and Laminin (20 μg/ml, Sigma). Calcium indicator Fura-2, a fluorescent dye that switches its excitation peak from 340 to 380 nm when bound to calcium, allows the concentration of intracellular calcium to be determined based on the ratio of fluorescence emission after sequential excitation at 340 and 380 nm (Grienberger and Konnerth, 2012). Cells were preloaded with 5 μM Fura-2 AM (Invitrogen) in Krebs solution (132 mM NaCl, 4 mM KCl, 1.4 mM MgCl_2_, 2.5 mM CaCl_2_, 6 mM glucose, 10 mM HEPES, pH 7.4) for 45 min at 37°C in an incubator with 5% CO_2_ and 95% atmospheric air. Dishes were washed in Krebs solution and then mounted on an inverted microscope with epifluorescence optics (Axiovert 135TV, Zeiss). Cells were continuously perfused with Krebs solution and stimulated by applying high-potassium Krebs solution (containing 10–100 mM KCl, isosmotic substitution with NaCl), or 100 μM histamine. Ratio images were obtained from image pairs acquired every 200 ms by exciting the cells at 340 nm and 380 nm. Excitation wavelengths were changed through a high-speed switcher (Lambda DG4, Sutter Instrument, Novato, CA, United States). The emission fluorescence was recorded at 510 nm by a cooled CDD camera (Photometrics CoolSNAP fx). Images were processed and analyzed using the software MetaFluor (Universal Imaging, West Chester, PA, USA). Regions of interest were defined manually.

### Heatmaps

For hierarchical clustering, ClustVis analysis software^1^ was used with the rows clustered using correlation distance and average linkage (Metsalu and Vilo, 2015).

### Statistical Analysis

Statistical analysis was done in Graphpad. Error bars represent the standard error of the mean (SEM). When appropriate, statistical analysis was done using a two-tailed *t*-student test for independent samples, and a *p*-value of less than 0.05 was considered statistically significant.

## Results

### Efficient Neural Commitment of hiPSCs and Exposure to Cannabinoids

**Figure 1:**
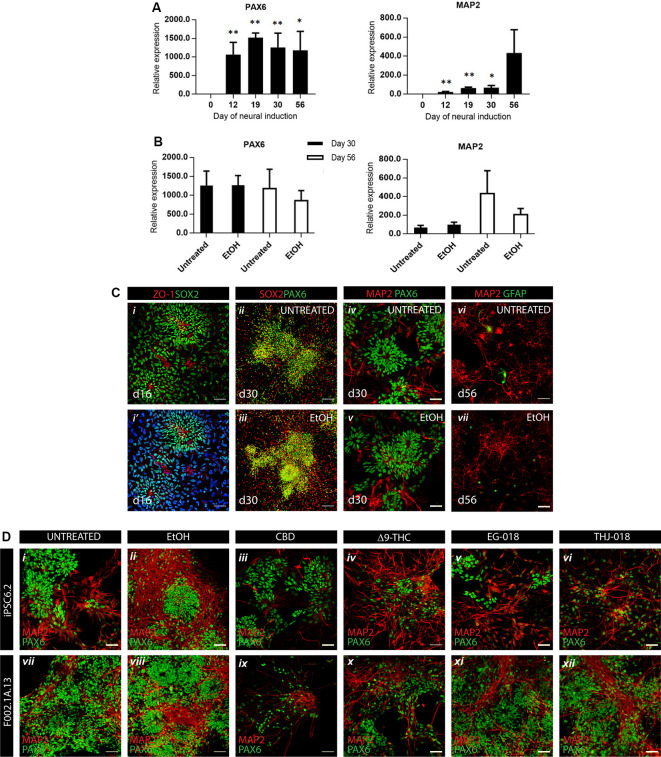
Efficient neural differentiation of hiPSCs and the effect of cannabinoid exposure. **(A)** qRT-PCR analysis of neural progenitor (*PAX6*) and neuronal (*MAP2*) mRNA expression levels relative to *GAPDH* at indicated timepoints. Data were analyzed by unpaired *t*-test, *p < 0.05, **p < 0.01; error bars represent standard error of the mean (SEM). **(B)** qRT-PCR analysis of *PAX6* and *MAP2* mRNA levels in untreated vs. vehicle-treated (0.01% EtOH) cultures showing no significant differences by unpaired *t*-test. Data in panels **(A,B)** were obtained from four independent experiments using iPSC6.2 cells. **(C)** Immunofluorescence for neural progenitor, neural and glial markers at different timepoints showing efficient neural commitment and differentiation of hiPSCs. Scale bars in panels **(i,i’)**, 100 μm. Scale bars in panels **(ii–vii)**, 50 μm. **(D)** Immunofluorescence at day 30 for neural progenitor marker PAX6 and neuron-specific microtubule-associated protein MAP2 in untreated (i, vii), vehicle-treated (ii, viii), and exposed to cannabinoids from day 19 to day 30 cultures (iii–vi, ix–xii), in two different iPSC lines, iPCS6.2 (male donor) and F002.1A.13 (female donor). Scale bars: 50 μm.

Neural differentiation of hiPSCs was achieved by controlled aggregation in serum-free medium N2B27 in the presence of SB431542, an inhibitor of TGFβ signaling, and LDN193189, an inhibitor of BMP signaling, both necessary for the acquisition of neuroepithelial identity (Chambers et al., 2009). Aggregates were cultured in non-adherent conditions until day 12 and then re-plated on laminin-coated plates to allow for the proliferation of neuroepithelial progenitors, further supported by the addition of bFGF between days 13 and 15, as depicted on the scheme of the experiment (Supplementary Figure S1). More than 1,000-fold increase in the expression of neural progenitor gene *PAX6* was consistently registered from day 12 on (Figure 1B). On day 16, an efficient neural commitment was evidenced by the presence of numerous neural rosettes (Figure 1Ci), typical morphology of neural precursors growing in 2D conditions (Abranches et al., 2009). By day 19 these cultures reached confluency and were gently replated, without dissociation of individual rosettes. Further neuronal differentiation led to the expression of neuronal-specific gene MAP2 from day 19 on (Figure 1B) and to the appearance of glutamatergic VGLUT1 and GABAergic GAD67 markers on day 56 (Supplementary Figures S2A,B). On day 30 cultures contained numerous SOX2^+^/PAX6^+^ neural rosettes (Figures 1Cii,iii). that were still present at day 56 surrounded by MAP2^+^ neurons and very rare occasional GFAP^+^ glial cells (Figures 1Civ–vii).

After replating on day 19, and to mimic continuous prenatal exposure of developing CNS cells to cannabinoids, solutions of CBD, Δ^9^-THC, EG-018, and THJ-018 in EtOH were added to culture media at every medium change, to a final concentration of 10 μM, except CBD, which was added to a final concentration of 1 and 10 μM. The culture medium containing these substances was changed every other day between days 19 and 30. Cannabinoids were added to the cultures at every medium change until day 56, and at this point, all cultures were processed for analyses. EtOH-treated cultures (0.01%, vehicle control, Figures 1Ciii,v,vii, 1Dii,viii) showed some increase in neuronal marker MAP2 staining as compared with the untreated cells (Figures 1Cii,iv,vi,Di,vii) although this increase, also detected by qRT-PCR, was not significant and was not accompanied by changes in progenitor parker PAX6 (Figure 1B).

### Neurotoxicity of CBD at 10 μM Concentration

In two independent experiments, the addition of CBD at 10 μM concentration showed massive cell death upon second medium change, between days 21–22 in culture. In all further experiments, 1 μM of CBD was used. At this concentration, CBD-treated cultures did not differ from the controls by cell morphology and the presence of neural progenitor and neuronal markers (Figure 1D) although the density of CBD-treated cultures was systematically lower than in other conditions indicating a possible negative effect on proliferation and/or survival of neuronal progenitors and differentiated neurons.

### Exposure to Δ^9^-THC and SCs Promotes Neuronal Differentiation

**Figure 2:**
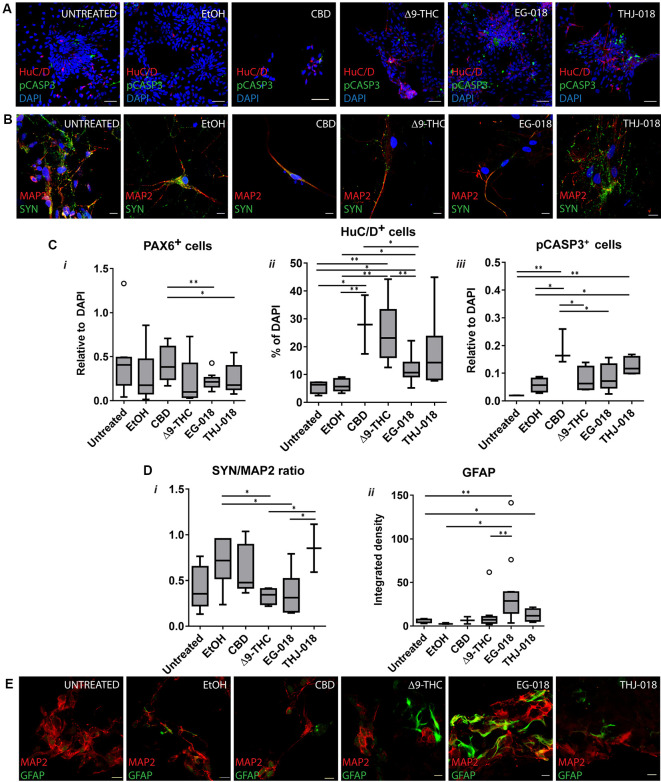
Effect of cannabinoid exposure on day 30 and 56 of neural differentiation.** (A)** Immunofluorescence for newborn neuronal marker HuC/D and apoptosis marker pCASPASE3 (pCASP3) at day 30 showing an increase in HuC/D staining and apoptotic cells in s cannabinoid-treated cultures. Scale bars: 15 μm. **(B)** Immunofluorescence for neuronal (MAP2) and synaptic protein synaptophysin (SYN) in cultures continuously exposed to cannabinoids from day 19 to day 56. Scale bars: 15 μm. **(C)** Quantification of PAX6^+^ **(i)**, HuC/D^+^ **(ii)** and pCASP3^+^ **(iii)** cells at day 30, relative to DAPI. Results for three independent experiments. **(D)** Quantification of the ratio of fluorescence intensity of SYN and MAP2 in day 56 cultures **(i)**, and of integrated fluorescence density for GFAP **(ii)**. Data from three independent experiments, 3–10 images per condition. Data in **(C)** and **(D)** analyzed by unpaired *t*-test; *p < 0.05, **p < 0.01; error bars represent SEM. Tukey’s range test was applied to determine outlier data points (open circles). **(E)** Immunofluorescence for glial (GFAP) marker and MAP2 in cultures continuously exposed to cannabinoids from day 19 to day 56. Scale bars: 15 μm.

By day 30 all six conditions showed expression of progenitor parker PAX6 and neuron-specific microtubule-associated protein 2 (MAP2; Figure 1D). Δ^9^-THC, EG-018, and THJ-018 -treated cultures showed decreased staining for the PAX6 neural rosette marker that was not reflected at the transcriptional level (Supplementary Figure S2C) suggesting a possible decrease of progenitor pool and exit for differentiation. However, transcript levels for neuron-specific gene *MAP2* showed a significant increase by day 30 only in THJ-018 condition (Supplementary Figure S2D) and immunostaining for this marker even decreased in EG-018-treated cultures (Supplementary Figure S3). To quantify better this effect, we performed immunostaining for HuC/D protein marker that is expressed earlier in differentiating CNS neurons (Figure 2A; Okano and Darnell, 1997; Abranches et al., 2009) and counted the percentage of cells expressing this antigen. Our data show that the decrease in the PAX6 staining (Figure 2Ci) is accompanied by the trend for an increased percentage of HuC/D^+^ cells upon exposure to all cannabinoids, with statistical significance in the case of THJ-018 (*p*-value < 0.0275; Figure 2Cii). In parallel, all CB-treated cultures exhibited elevated levels of apoptosis marker cleaved CASPASE3 (Figure 2Ciii), while there were no significant differences in the number of Ki-67^+^ proliferative cells (data not shown). Together, these results indicate that both Δ^9^-THC and two SCs lead to premature differentiation of rosette progenitors that seems to be more pronounced in the case of THJ-018, possibly due to lower neuronal survival upon exposure to Δ^9^-THC and EG-018. One of the reasons for lower neuronal survival could be a functional impairment or inability to achieve functional maturation.

### Exposure to Cannabinoids Leads to the Formation of Functionally Impaired Neurons

After detecting an increase in the number of differentiating neurons upon continuous exposure to cannabinoids we next questioned if these neurons were able to achieve functional maturation. For this goal, all cultures were maintained in BrainPhys^TM^ neuronal maturation medium for 16 days, with 1/3 medium volume change three times per week and continuous exposure to cannabinoids. Immunofluorescent staining for mature synaptic protein SYN of Δ^9^-THC and EG-018-treated day 56 cultures revealed decreased staining intensity (Figures 2B,Di) suggesting a lower density of mature synaptic puncta. Additionally, an increase in glial acidic fibrillary protein (GFAP) staining was visible in both conditions (Figure 2E), indicating premature glial differentiation, which is detectable in untreated cultures only around day 80 (not shown) and was found to be increased in Δ^9^-THC and SCs-exposed cultures (Figure 2Dii). To further evaluate both the neuron-glia ratio and the functionality of these cultures we performed SCCI. Control and CB-treated cultured cells were sequentially stimulated by exposure to KCl and histamine. Differentiated functional neurons are expected to open voltage-sensitive calcium channels in response to KCl, resulting in a massive influx of calcium to the cytoplasm (Ambrósio et al., 2000; Macías et al., 2001). Immature neurons, neural progenitors and glial cells express functional histamine receptors, which stimulation also increases intracellular calcium concentration. Indeed, histamine/KCl ratios can be used to evaluate the proportion of mature/immature neurons in these cultures (Agasse et al., 2008; Rodrigues et al., 2017).

**Figure 3:**
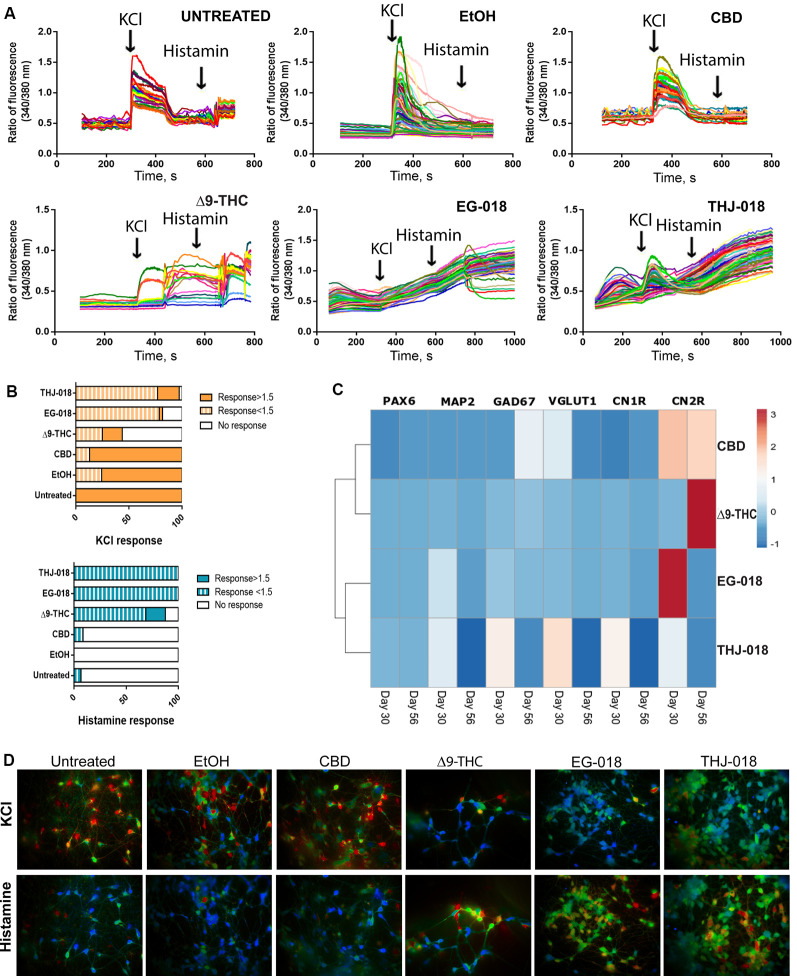
Functional assessment of cannabinoid-treated cultures on day 56. **(A)** Single-cell calcium imaging (SCCI) analysis of day 56 cultures showing abnormal response to KCl and histamine stimuli by Δ^9^-THC, EG-018, and THJ-018-treated neuronal cells. **(B)** Summary of SCCI analyses presented as percentage of responding and non-responding cells for KCl and histamine stimulation. The timepoint of response corresponds to the peak seen in the graph in panel **(A)**, and the ratio of fluorescence value for this timepoint over baseline level >1 was considered as a response. For EG-018 and THJ-018 conditions, the last time point before washing was used to calculate the response to histamine stimulation. **(C)** Hierarchical clustering illustrates relative expression levels of different genes at day 30 and 56 of neural differentiation. *PAX6, MAP2, GAD67, VGLUT1, CNR1*, and *CNR2* were analyzed by qRT-PCR. Rows are centered; unit variance scaling is applied to rows. Rows are clustered using correlation distance and average linkage. Corresponding qRT-PCR data are presented in Supplementary Figure S2. **(D)** Single-cell calcium imaging. Representative ratio images for different culture conditions on day 56. Images were taken immediately after cells received the indicated stimulus (KCl or histamine).

Upon KCl stimulation, a sharp increase in cytosolic calcium concentration was observed in both controls and CBD-treated cultures (Figures 3A,D), with an average fold change of fluorescence intensity around two in all three conditions (Figure 3B, Supplementary Figure S2I). In contrast, very few cells responded to histamine in these cultures, and the few responding cells exhibited fold change below 1.5 (Figure 3B, Supplementary Figures S2J,K), indicating that most of the cells in these cultures are excitable neurons, with a small proportion of neural progenitors/glia. These results agree with very rare occasional GFAP staining detected in both control cultures at this stage, contrasting with numerous GFAP^+^ cells in Δ^9^-THC and SC-treated cultures.

Exposure to CBD has a small but statistically significant effect on the functionality of differentiating neuronal cells, with a lower amplitude of response to KCl stimulation compared with the controls (Figure 3A; Supplementary Figure S2I). In sheer contrast to this, the exposure to both Δ^9^-THC and SCs severely impaired the ability to differentiate neurons to respond to both KCl and histamine stimulation (Figures 3A,B). Δ^9^-THC-treated cells showed delayed (85 s vs. 28–35 s in control conditions) response to KCl, with 56.3% of non-responding cells contrasting with the absence of non-responding cells in both controls (Supplementary Figure S2K). As the majority of KCl-responding cells either responded too late or were unable to return to baseline intracellular calcium levels upon KCl stimulation, it is impossible to determine the exact proportion of cells that responded to histamine. Therefore, the higher proportion of histamine-responding cells (87.5% vs. 6.9% in untreated control and 0% in EtOH-treated cultures, Supplementary Figure S2K) most probably corresponds to a mixture of functionally impaired neuronal cells showing abnormal response to KCl stimulation with some glia-like GFAP^+^ cells that are also more abundant in this condition.

In comparison to Δ^9^-THC, THJ-018 has a slightly less severe effect on the functionality of neuronal cells, with less pronounced delay in response to KCl, 51 s vs. 28–35 s in control conditions. The percentage of KCl-responding cells is similar to the controls, 97.2%, however, the majority of responses, 76.9%, are below 1.5x fold increase (Supplementary Figure S2). Moreover, only 8.7% of cells exhibit a well-defined peak of response to KCl while 88.5% of cells show continuously increasing Ca^2+^ levels, being unable to return to baseline levels after washout of KCl-containing medium. The percentage of histamine-responding cells in this condition is even higher than that of Δ^9^-THC, reaching 100% of all assayed cells (Figure 3A, Supplementary Figure S2K), however, it is not supported by the increase in GFAP^+^ cells and includes almost all cells that already responded to KCl. Therefore, this response to histamine most probably represents that of functionally impaired neurons with very slow kinetics of KCl response, rather than that of neural progenitors or immature neurons expressing both voltage-gated calcium channels and histamine receptors (in the latter case, the well-defined peak of histamine response should also be present).

EG-018-treated cells represent the most severely affected condition (Figures 3A,B), with no discernible peak of KCl response and the highest percentage of cells with weak (<1.5x) or no response to KCl (78.8% and 18.2%, respectively, Supplementary Figure S2K). Like the THJ-018-treated cells, 100% of EG-018-treated cells seemingly responded to histamine, however, instead of a well-defined peak of the response, a continuous increase in intracellular Ca^2+^ was also observed in this case (Figure 3A). These data might indicate that exposure to EG-018 leads to an even greater delay in the cellular response to KCl stimulation. This conclusion is further supported by the absence of a well-defined peak of histamine response despite the abundance of GFAP^+^ cells in this condition (Figures 2Dii,E).

### RT-PCR Analysis of the Expression of Cannabinoid Receptors 1 and 2 and Neural Markers

qRT-PCR expression data for all four cannabinoids were normalized to EtOH condition (Supplementary Figures S2C–H) and analyzed by hierarchical clustering (Figure 3C). The most pronounced fold changes of expression levels were detected for the genes encoding cannabinoid receptors 1 and 2, *CNR1*, and *2*. Exposure to CBD, Δ^9^-THC, EG-018, and a less extent, THJ-018 led to a significant increase in the expression levels of *CNR2* (Figure 3C). Interestingly, levels of *CNR1* were slightly increased after exposure to Δ^9^-THC and two SCs by day 30 and decreased by day 56 (Figure 3C, Supplementary Figure S2G) probably reflecting the long-term downregulation of CB_1_ levels, similarly to what was reported to occur at both protein and mRNA level in a response to chronic exposure to Δ^9^-THC (Oviedo et al., 1993; Romero et al., 1997; Villares, 2007). However, both SCs and THJ-018 in particular were able to elicit a stronger effect than Δ^9^-THC.

Curiously, THJ-018 and CBD, which were found to cause less functional impairment on neuronal cells according to SCCI data, led to more pronounced changes in gene expression levels in both neural markers and CB receptor genes. Thus, the effect of exposure to these two substances requires further examination by different techniques than those used in this study, to uncover the nature of the cellular response to these substances.

## Discussion

The recreational use of cannabis is being legalized in an increasing roll of countries and as many as 57% of adults in the USA favor this tendency (Harris and Okorie, 2017). Not surprisingly, the rates of cannabis use show a consistent increase over past years, inclusively in pregnant and non-pregnant women (Brown et al., 2017; Harris and Okorie, 2017). Alarmingly, in a recent study, 19% of 18–24-year aged pregnant women screened positive for marihuana, showing a trend to increase in the last decade (Young-Wolff et al., 2017). The outcome of prenatal exposure to cannabinoids on human neurodevelopment can be evaluated only retrogradely, after several years or even decades, through the assessment of cognitive, motor, and behavioral scores. A plethora of other intervening factors introduces huge variations of the outcome, making it difficult to conclude which are the direct consequences of prenatal exposure to cannabis (reviewed in Wu et al., 2011; Scheyer et al., 2019). In this work, we propose a simplified system that reproduces the initial steps of neural differentiation from human pluripotent cells, where we expose neural cells to cannabinoids in a continuous way, mimicking the regular, three-times per week usage. The concentration of 10 μM used for Δ^9^-THC, THJ-018, and EG-018, corresponds to 314, 342, and 391 ng/ml, respectively. As an example, smoking a joint with 3, 55% of Δ^9^-THC leads to a plasma peak concentration of 150 ng/ml after 10 min (Huestis, 2007). However, the Δ^9^-THC concentration of current marijuana can reach 20% which can lead to a plasma peak concentration of Δ^9^-THC higher than 800 ng/ml. Thus, the concentration of Δ^9^-THC used in this work is similar to smoking a joint with 7% of Δ^9^-THC, corresponding to the CB_1_ saturation level of 70–80%. In our system, the addition of 10 μM CBD was neurotoxic, while 1 μM concentration consistently yielded low culture densities possibly also due to neurotoxicity. However, exposure to 1 μM CBD led to an increase in GABAergic and decrease in glutamatergic markers expression levels (Figure 3C, Supplementary Figures S2E,F) hinting at a possible disbalance between the number of excitatory and inhibitory neurons that needs to be further investigated. CBD concentrations between 1 μM and 14 μM were found not to be cytotoxic to HUVEC cells (Solinas et al., 2012), while 10 μM CBD showed no toxicity for human breast carcinoma (Namdar et al., 2019). The same concentration of CBD was neurotoxic in our model. The neurotoxicity of CBD is of particular concern given that CBD content can reach 25% in several legally available cannabis preparations with reported blood concentration reaching 82.6 ng/ml (0.263 μM) after chronic use (Meier et al., 2018), which is a just a small fraction of the total CBD concentration in the body of these users. The ratio between the concentration of a lipophilic drug in the fat tissue and plasma at a steady-state can reach a value of 3–4 digits undermining the measured drug concentration found by blood analysis. Δ^9^-THC is a partial agonist of CB_1_ and many of its effects on CNS were shown to be mediated by CB_1_ (Pertwee, 2008). CB_1_ antagonist SR1411716 was shown to increase neuronal differentiation (Rueda et al., 2002), while CB_1_ KO decreased progenitor proliferation (Aguado et al., 2005). In our study, chronic exposure to Δ^9^-THC promoted neuronal (Figures 1D, 2C, Supplementary Figure S2) and glial (Figures 2Dii,E) differentiation, resembling the effect of CB_1_ antagonist. However, the mRNA levels of *CNR1* decreased only slightly in this condition (Supplementary Figure S2G), while those of *CNR2* increased (Supplementary Figure S2H). The interplay of CB_1_ and CB_2_ was implicated in the modulation of postnatal neurogenesis in rodents (Rodrigues et al., 2017). In our study, exposure to Δ^9^-THC and SCs differentially impacted the expression levels of *CNR1* and *CNR2* also supporting the view that both receptors might be involved in the neurogenesis. In a recent study, RNA transcriptomic analyses of hiPSC-derived neurons exposed both acutely and chronically (for 7 days) to 1 μM Δ^9^-THC revealed significant changes in genes associated with intellectual disability, autism and psychiatric disorders (Guennewig et al., 2018). Interestingly, they showed that chronic Δ^9^-THC exposure resulted in the downregulation of several histone-binding genes including *MECP2*, Rett syndrome causing genes. The lack of this gene results in precocious neuronal and glial differentiation in a forebrain organoid model of the disease, similarly to our results of chronic exposure to Δ^9^-THC. A different study employed continuous exposure to eCB AEA and Δ^9^-THC in dopaminergic neuronal differentiation from hiPSCs (Stanslowsky et al., 2017). These authors show that 10 μM concentrations of both cannabinoids impaired neuronal function by reducing voltage-gated sodium and potassium currents, action potential amplitudes and spontaneous synaptic activity. Our data further support functional impairment induced by exposure to Δ^9^-THC, EG-018 and THJ-018, by demonstrating the inability of CB-treated neurons to increase their intracellular Ca^2+^ levels in response to KCl stimulus. SCs used in this study are Novel Psychoactive substances with properties similar to Δ^9^-THC exhibiting considerably higher binding affinities to CB receptors (Hess et al., 2016; Schoeder et al., 2018). THJ-018 is 2^nd^ generation SC behaving as a slightly better than Δ^9^-THC partial agonist of both CB_1_ and CB_2_ in cAMP accumulation essay (Hess et al., 2016). In contrast, EG-018 was shown to activate CB_1_ more than full agonist CP55, 940, having much less activity on CB_2_ (Schoeder et al., 2018). This differential receptor activation capacity of the two SCs might explain some of the differences observed in this study. Generally stronger effect of EG-018 exposure might be due to its higher capacity to activate CB_1_. However, experiments using selective receptor agonists and antagonists should be conducted to unveil the mechanisms of EG-018 action.

The observed functional impairment induced by chronic exposure to Δ^9^-THC, EG-018, and THJ-018 during neuronal differentiation and formation of functional neuronal circuitry might help to explain the observed link between prenatal exposure to cannabis and psychiatric disorders. Δ^9^-THC-treated neurons displayed synaptic and glutamate signaling alterations resembling those observed in schizophrenia patient iPSC-derived neurons (Guennewig et al., 2018). Another interesting avenue to explore in future studies is the observed difference in CB-induced phenotype severity between two iPSC lines, with male cell line iPSC6.2 being more affected by Δ^9^-THC and SCs than the female F002.1A.13 (Figure 1D). Sex-dependent susceptibility to Δ^9^-THC has been reported before and in a recent study using a mouse model of prenatal exposure male offspring was particularly affected showing pronounced hippocampal interneuronopathy (de Salas-Quiroga et al., 2020).

In conclusion, our data show that continuous exposure to both Δ^9^-THC and SCs can induce functional impairment to newborn neurons during the formation of the human CNS, which is able to produce a deep and lasting impact on the overall brain structure and functioning. By showing this impairment, our data contribute to support the observations of long-lasting alterations in neural activity in adolescents subjected to prenatal marihuana exposure (Smith et al., 2004; Wu et al., 2011; Grant et al., 2018).

## Data Availability Statement

All datasets generated for this study are included in the article/Supplementary Materials.

## Author Contributions

EB and AQ outlined the general framework of this study. CM, EB, AQ, and CF designed the experiments. CM, EB, SV, and TB performed the experiments. CF and AQ provided the unique reagents. CM and EB analyzed the data. EB and CM wrote the manuscript.

## Conflict of Interest

The authors declare that the research was conducted in the absence of any commercial or financial relationships that could be construed as a potential conflict of interest.

## Footnotes

^1^http://biit.cs.ut.ee/clustvis

## Supplementary Material

FIGURE S1 A scheme representing culture procedure to induce neural differentiation of hiPSCs showing culture media and supplements used as well as cell morphologies observed at different time points. Individual experiments, cell lines, and substances used are depicted below the scheme. Solid green boxes represent total medium change every other day, while striped green boxes show the 1/3 medium change three times a week.

FIGURE S2 **(A,B)** qRT-PCR analysis of *GAPDH*-normalized expression levels of GABAergic neuronal marker *GAD67* **(A)** and glutamatergic marker *GLUT1* **(B)** along with the differentiation in untreated cultures. **(C–H)** qRT-PCR analysis of relative expression levels of neural progenitor (*PAX6*, **C**), neuronal (*MAP2*, **D**), GABAergic (*GAD67*, **E**) and glutamatergic (*GLUT1*, **F**), and CB receptors *CNR1* **(G)** and CNR2 **(H)**. *GAPDH*-normalized expression levels were further normalized against EtOH (vehicle) condition. All qRT-PCR data analyzed by unpaired *t*-test; **p* < 0.05, ***p* < 0.01; error bars represent SEM. **(I–J)** Quantification of the SCCI data regarding the strength of the response to KCl **(I)** and histamine **(J)**. The value of the response was considered at the time point corresponding to the peak seen in the graph in Figure 3A, and the ratio of fluorescence value for this timepoint over baseline level was calculated for each responding cell. For EG-018 and THJ-018 conditions, the last time point before washing was used to calculate the response to histamine stimulation. Data analyzed by unpaired *t*-test; **p* < 0.05, ***p* < 0.01; error bars represent SEM. **(K)** Values used for building the graphs in Figure 3B. Percentages calculated as described in the legend for Figure 3.

FIGURE S3 Quantification of MAP2^+^ cells at day 30, relative to DAPI. Results from three independent experiments. Tukey’s range test was applied to determine outlier data points (open circles). Data analyzed by unpaired *t*-test; **p* < 0.05, ***p* < 0.01; error bars represent SEM.
