# Supplementary figures and images for: hiPSC-Based Model of Prenatal Exposure to Cannabinoids: Effect on Neuronal Differentiation
Source: Front Mol Neurosci. 2020 Jul 6;13:119. doi: 10.3389/fnmol.2020.00119 (PMC7357827; doi:10.3389/fnmol.2020.00119)

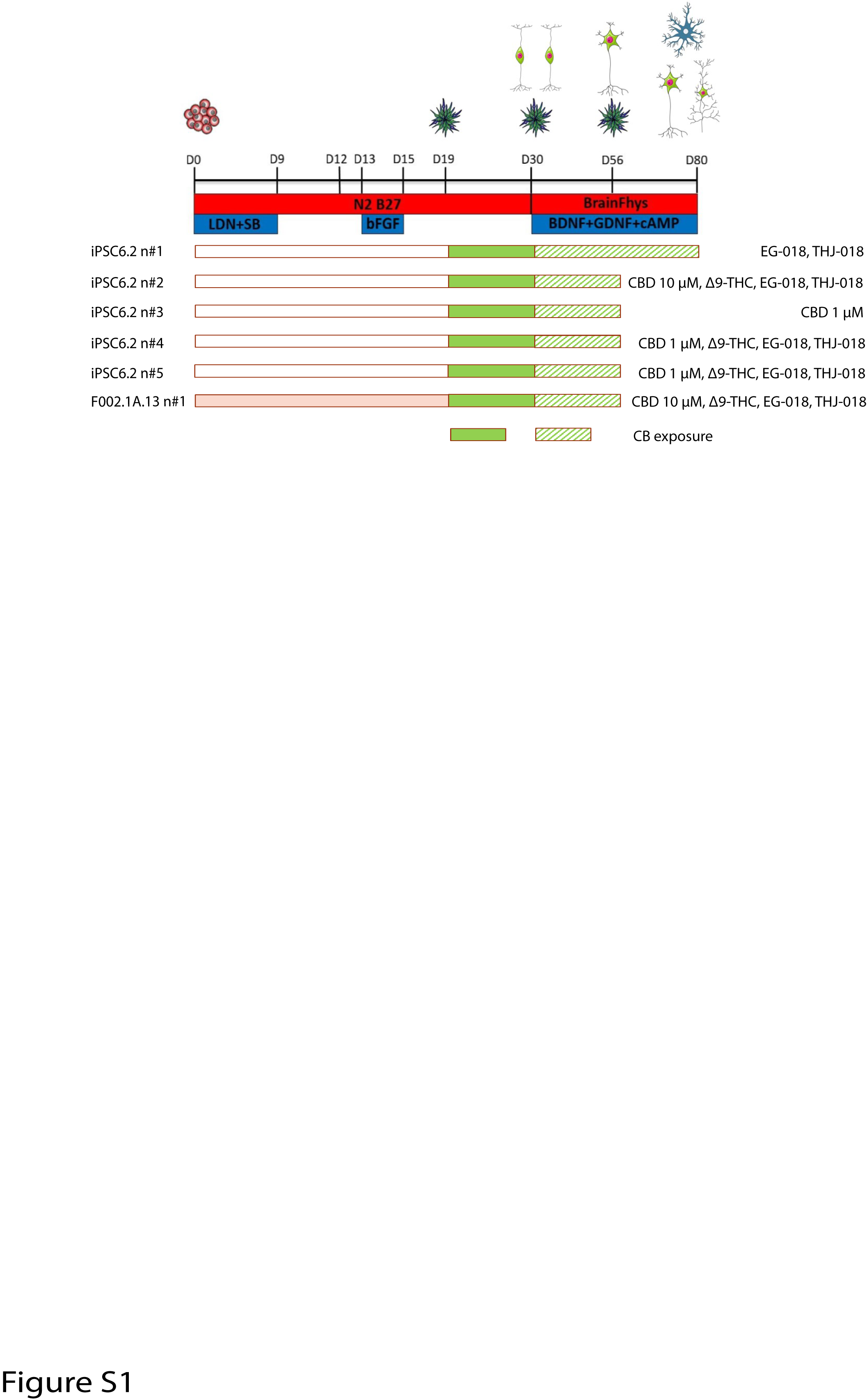

Supplement: FIGURE S1 — A scheme representing culture procedure to induce neural differentiation of hiPSCs showing culture media and supplements used as well as cell morphologies observed at different time points. Individual experiments, cell lines, and substances used are depicted below the scheme. Solid green boxes represent total medium change every other day, while striped green boxes show the 1/3 medium change three times a week. [file Image_1.TIF]

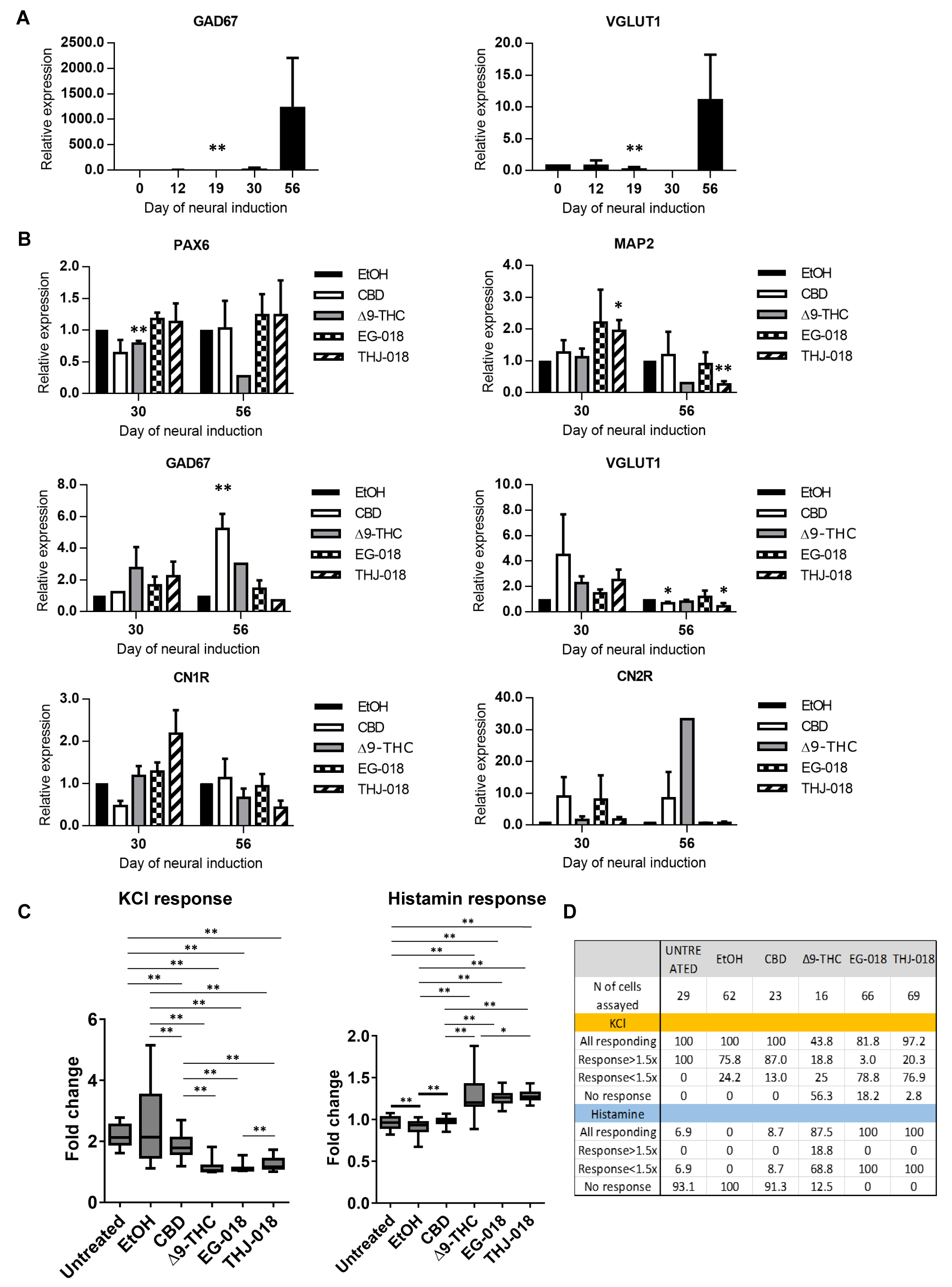

Supplement: FIGURE S2 — (A,B) qRT-PCR analysis of GAPDH-normalized expression levels of GABAergic neuronal marker GAD67 (A) and glutamatergic marker GLUT1 (B) along with the differentiation in untreated cultures. (C–H) qRT-PCR analysis of relative expression levels of neural progenitor (PAX6, C), neuronal (MAP2, D), GABAergic (GAD67, E) and glutamatergic (GLUT1, F), and CB receptors CNR1 (G) and CNR2 (H). GAPDH-normalized expression levels were further normalized against EtOH (vehicle) condition. All qRT-PCR data analyzed by unpaired t-test; *p < 0.05, **p < 0.01; error bars represent SEM. (I–J) Quantification of the SCCI data regarding the strength of the response to KCl (I) and histamine (J). The value of the response was considered at the time point corresponding to the peak seen in the graph in Figure 3A, and the ratio of fluorescence value for this timepoint over baseline level was calculated for each responding cell. For EG-018 and THJ-018 conditions, the last time point before washing was used to calculate the response to histamine stimulation. Data analyzed by unpaired t-test; *p < 0.05, **p < 0.01; error bars represent SEM. (K) Values used for building the graphs in Figure 3B. Percentages calculated as described in the legend for Figure 3. [file Image_2.TIF]

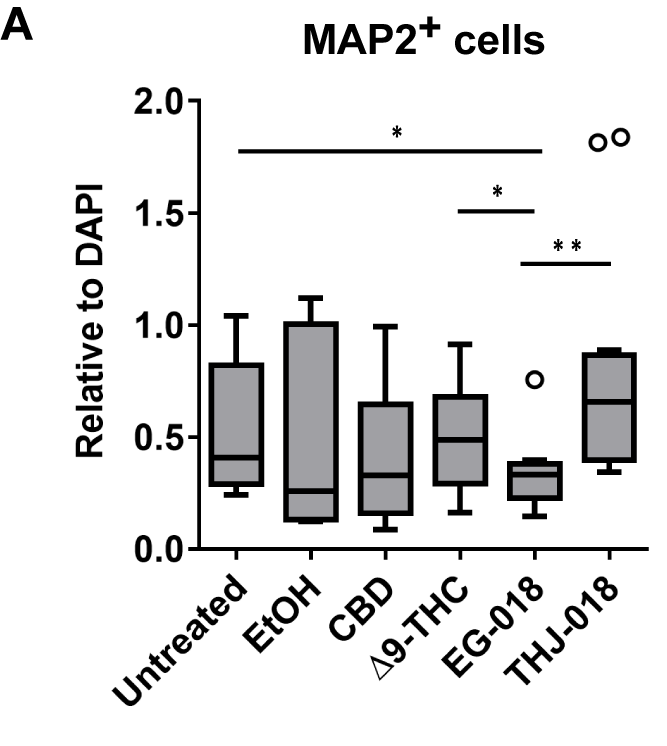

Supplement: FIGURE S3 — Quantification of MAP2+ cells at day 30, relative to DAPI. Results from three independent experiments. Tukey’s range test was applied to determine outlier data points (open circles). Data analyzed by unpaired t-test; *p < 0.05, **p < 0.01; error bars represent SEM. [file Image_3.TIF]
